# A most aggressive bear: Safari videos document sloth bear defense against tiger predation

**DOI:** 10.1002/ece3.11524

**Published:** 2024-07-11

**Authors:** Thomas R. Sharp, David L. Garshelis, Wesley Larson

**Affiliations:** ^1^ Wildlife SOS Salt Lake City Utah USA; ^2^ International Union for Conservation of Nature, Species Survival Commission Bear Specialist Group Gland Switzerland; ^3^ Cohasset Minnesota USA; ^4^ Lolo Montana USA

**Keywords:** bear attacks on humans, behavioral adaptations, citizen science, defensive aggression, *Melursus ursinus*, myrmecophagy, online videos, *Panthera tigris*

## Abstract

Sloth bears are non‐carnivorous yet they attack more people than any other bear. They often stand up and charge explosively if a person mistakenly gets too close. Here, we argue that their aggression toward humans is an extension of their behavior toward tigers, which are their only natural predator. Interactions between sloth bears and tigers have not previously been studied because scientists have rarely observed such events. We collected and examined 43 videos or photo documentations of sloth bear–tiger interactions posted on the internet or social media from 2011 to 2023, mainly by tourists visiting tiger parks in India. We observed that sloth bears were most likely to stand up and charge if they first became aware of the tiger at close range (<3 m away). This aggressive–defensive strategy, intended to dissuade the tiger from attacking, appeared to be successful, in that 86% of interactions ended with no contact, whereas four (9%) culminated in the bear's death. We propose that a myrmecophagous diet led to this species' aggressive behavior: (1) their long, blunt front claws, well adapted for digging termites and ants, hamper their ability to climb trees for escape, and (2) they walk with their head down focused on scents underground, and make considerable noise digging and blowing soil, enabling tigers to approach quite closely without being detected. Sloth bears have coexisted with tigers or other (now extinct) large felid predators for their entire evolutionary history. Whereas their aggressive behavior has served them well for millions of years, more recently, people's fear of and retaliation against sloth bears represents a major threat to their survival. Understanding how sloth bears react to tigers provides guidance for reducing attacks on humans, thereby contributing to sloth bear conservation. Our investigation was made possible by passive citizen scientists, who unknowingly collected valuable data.

## Graphical abstract

We collected and examined 43 videos, and photo documentations, of sloth bear–tiger interactions posted on the internet or social media mainly by tourists visiting tiger parks in India. We observed that sloth bears were most likely to stand up and charge if they first became aware of the tiger at close range (<3 m away). This aggressive–defensive strategy, intended to dissuade the nearby tiger from attacking, appeared to be successful, in that 86% of interactions ended with no contact, whereas four (9%) culminated in the bear's death. The photographer of the photo in the graphical abstract is Vikram Hiresavi.

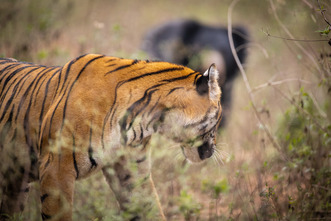

## 1 INTRODUCTION

Sloth bears (*Melursus ursinus*) are believed to be one of the most dangerous wild animals on the Indian subcontinent (Pillarisett, 1993; Silwal et al., 2017; Sterndale, 1884). They are well‐known for their propensity to attack humans, rushing quickly in a burst of energy, biting or clawing the victim's face, and causing serious, sometimes fatal injuries (Bargali et al., 2005; Debata et al., 2016; Dhamorikar et al., 2017; Garcia et al., 2016; Rajpurohit & Krausman, 2000; Ratnayeke et al., 2014; Sharp et al., 2020; Singh et al., 2018). Baker (1887: 222–223), who claimed 40 years of experience hunting a host of big game animals in India during the 1800s, considered sloth bears the “fiercest.” He commented: “Should an unarmed wayfarer meet Master Bruin [sloth bear] engaged in looking over his orchards, or sauntering over his domain, let him step aside silently lest he have his scalp drawn over his face, or his features so altered as to be unrecognizable by his most intimate friends.”

Rajpurohit and Krausman (2000) tallied 735 sloth bear attacks on people in just 5 years (115–185 per year, averaging nearly 150 per year) in Madhya Pradesh, India, of which 48 (almost 10 per year) were fatal. To put this in perspective, worldwide, across three continents and 44 range countries, brown bears (*Ursus arctos*) attack ~40 people per year, of which ~6 per year are fatal (Bombieri et al., 2019). Yet, brown bears (which include grizzly bears) are commonly thought of as a highly aggressive and feared bear species in many parts of their range (Bombieri et al., 2018), whereas sloth bears, which are responsible for a much higher number of attacks, have not gained a similar reputation, except on the Indian subcontinent. Tallying all large carnivore attacks on people worldwide between 1950 and 2019, Bombieri et al. (2023) reported that sloth bears far exceeded all other species, including tigers (*Panthera tigris*).

The aggressive nature of sloth bears toward humans is enigmatic, as the species is not a predator of mammals [although there are occasional cases of carrion in the diet; see review by Rabari and Dharaiya (2022)]. Sloth bears feed primarily on insects, especially termites and ants, as well as various seasonally available fruits (Baskaran et al., 2015; Joshi et al., 1997; Khanal & Thapa, 2014; Philip et al., 2021; Ramesh, Sankar, & Qureshi 2009; Rather et al., 2020; Seidensticker et al., 2011). Even in agricultural areas, where they eat some cultivated crops, they are not known to prey on livestock (Palei et al., 2020). Of the eight species of bears, sloth bears, along with giant pandas (*Ailuropoda melanoleuca*), are at the very low end of the spectrum of carnivory; however, whereas giant pandas are unaggressive toward humans, sloth bears are highly aggressive. This irony is captured in an old book by Glasfurd (1906), which describes the author's several encounters with aggressive sloth bears while hunting, and a drawing of the species aptly captioned “vegetarian and hypocrite.”

The aggressive nature of brown bears is more easily understood, as this species frequently preys on large mammals in parts of its range (see reviews by Bojarska & Selva, 2012; Niedziałkowska et al., 2019; Zager & Beecham, 2006). Brown bears also occasionally attempt to prey on people, and perhaps for that reason, the percent of attacks on people that result in fatalities is higher for brown bears [14% worldwide (Bombieri et al., 2019); >40% in Russia (Kudrenko et al., 2020, 2022)] than for sloth bears [7–8% (Bargali et al., 2005; Rajpurohit & Krausman, 2000; Sharp et al., 2020)]. However, the sheer number of sloth bear attacks is remarkable, especially for a species whose diet is primarily termites, ants, and fruits.

Encounter rates between humans and bears also affect the risk of bear attacks. Large human populations near bear habitat or high human use of bear habitats may lead to more bear‐inflicted human fatalities (Herrero et al., 2011). Given that roughly 90% of sloth bear range is in India (Dharaiya et al., 2020), where dense human populations live in villages adjacent to sloth bear habitat, it reasonably follows that sloth bear–human encounter rates are high. However, human behavior and bear behavior also strongly affect the outcome of an encounter, or whether their proximity even results in an encounter (i.e., whether the bear leaves before being noticed). Smith and Herrero (2018) highlighted species‐specific differences between brown bears and American black bears (*U. americanus*) in Alaska, USA, by examining records where people were at risk of being injured (or were injured or killed) by bears over a period of 135 years. They found that brown bears were involved in 88% of conflicts that threatened people, despite black bears being ~3x more abundant and also living in areas more frequented by people. These data indicate strong underlying behavioral differences between these two bear species in terms of their interactions with humans.

Here we posit that the aggressive nature of the sloth bear toward humans may be related to its evolved defensive behavior toward tigers and other (now extinct) large predators. Bengal tigers (*P. t. tigris*), the subspecies of the Indian subcontinent, may be twice the size and weight of a sloth bear, and represent a considerable threat. In some tiger parks in India, up to 2% of tiger scats include sloth bear remains (Biswas & Sankar, 2002; Reddy et al., 2004). While sloth bears are occasionally predated upon by tigers, they are also well known for surviving tiger encounters by aggressively fighting off their would‐be killers, as has been documented in various accounts since the 1800s (Brander, 1982; Cambell, 1894; Clutterbuck, 1894; Fenton, 1909; Gopal, 1991; Joshi et al., 1999; Laurie & Seidensticker, 1977; Littledal, 1889).

Until recently, data on sloth bear–tiger interactions have been difficult to obtain due to the scarcity of visual observations and scant photographic evidence that can be studied in detail. Though videos of these interactions are still rare, the proliferation of smart phones with video capability, and the ability and propensity of people to post videos to websites and social media, has made it possible to more effectively study these interactions and begin to make inferences about sloth bear aggressiveness. Here we collected publicly available photo and video documentations of sloth bear–tiger interactions, from which we discerned patterns of behavior, and related these behaviors to sloth bear ecology and evolution. These patterns of behavior between sloth bears and tigers provide a clearer understanding of why sloth bears behave as they do toward humans. Moreover, insights from this study help inform messaging about safety for people working, recreating, or living in sloth bear habitat.

## 2 STUDY AREA

**FIGURE 1:**
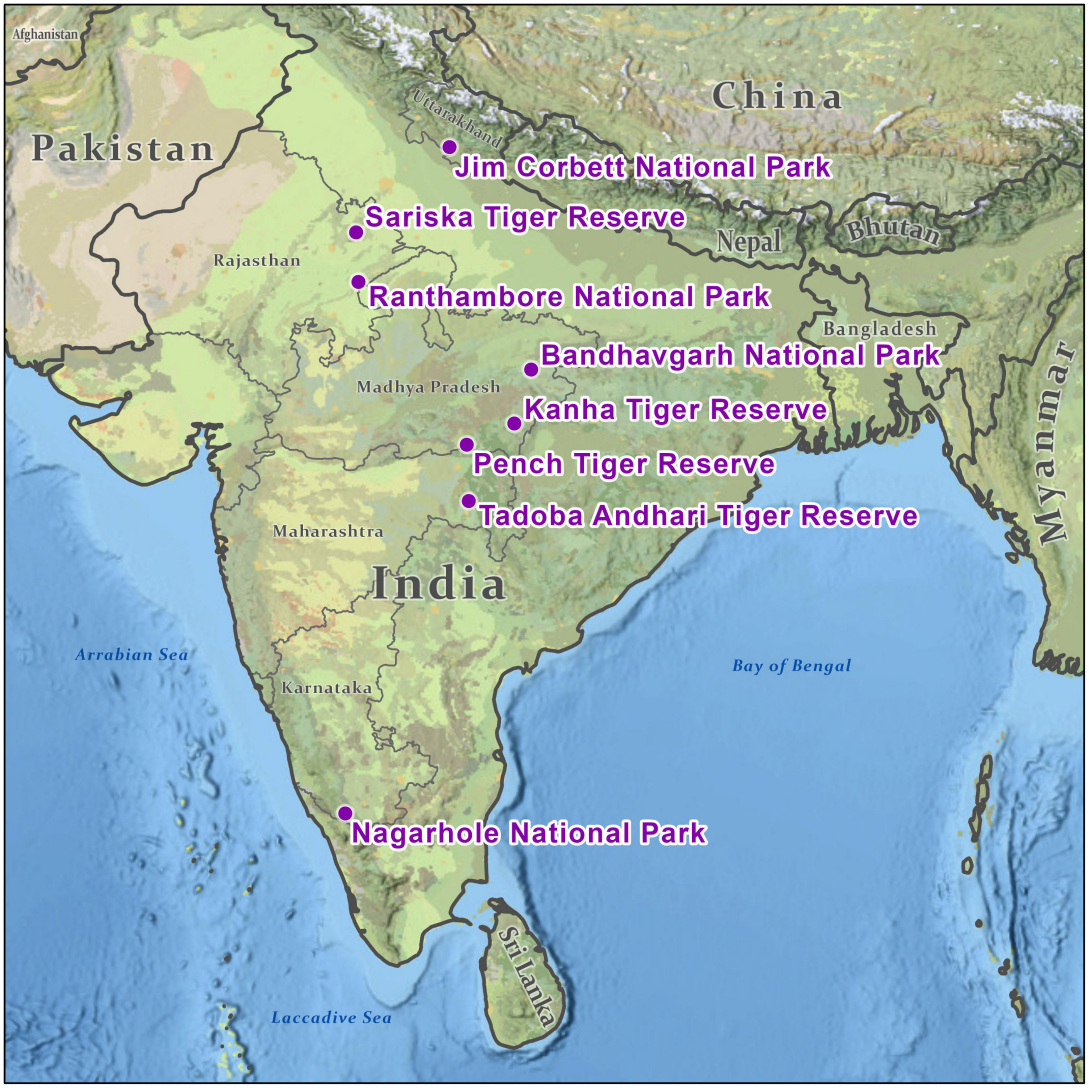
Locations of documented interactions between sloth bears and tigers in India.

Sloth bears and tigers once overlapped across much of the Indian subcontinent. Currently, they overlap to a large extent in tiger reserves or conservation units across India and southern Nepal. Video and photo‐documentations of sloth bear tiger interactions used in this study were collected in eight different tiger reserves across India, from latitudes between 29.55° N and 12.07° N, including Ranthambore National Park, Tadoba Andhari Tiger Reserve, Nagarhole National Park, Kanha Tiger Reserve, Pench Tiger Reserve, Bandhavgarh National Park, Sariska Tiger Reserve, and Jim Corbett National Park (Figure 1). Photo‐safaris in these parks are often centered around tigers.

Habitat types vary across these tiger reserves but are largely made up of tropical dry deciduous and moist deciduous forest. These reserves host a wide array of common and threatened wildlife species in addition to the sloth bear, listed as Vulnerable on the IUCN Red List, and tiger, listed as Endangered (Dharaiya et al., 2020; Goodrich et al., 2022). Other notable wildlife species found in these reserves include the Asian elephant (*Elephas maximus*), leopard (*Panthera pardus*), dhole (*Cuon alpinus*), striped hyaena (*Hyaena hyaena*), golden jackal (*Canis aureus*), spotted deer (*Axis axis*), sambar deer (*Rusa unicolar*), rhesus macaque (*Macaca mulatta*), gray langur (*Semnopithecus priam*), Indian gray mongoose (*Urva edwardsii*), Indian peafowl (*Pavo cristatus*), Indian Gray Hornbill (*Ocyceros birostris*), Common Kingfisher (*Alcedo atthis*), Green Bee Eater (*Merops orientalis*), mugger crocodile (*Crocodylus palustris*), Indian rock python (*Python molurus*), and common cobra (*Naja naja*).

## 3 METHODS

We searched the internet as well as social media sites for posted videos or sequences of still photos showing interactions between sloth bears and tigers. We conducted these searches regularly during 2020–2023 to find new postings. We used popular search engines (Google, YouTube, Yahoo, and Bing) and video search functions within those engines. All searches included the words *sloth bear* + *tiger*, and then narrowed with additional terms such as *interaction* and *fight*. We also searched Instagram and Facebook using the same terms. Videos and photos that we found were made by tourists, naturalists, and professional photographers, who, generally by happenstance, saw a sloth bear and tiger in proximity, or while photographing one species, the other species appeared. These videos were made from the safety of a vehicle or machan (viewing platform), and those that were posted may represent a selection that photographers thought were most interesting (i.e., would be viewed the most or gain the most engagement).

We defined a “sloth bear–tiger interaction” as situations when a tiger observed a sloth bear or a sloth bear observed a tiger or when the two species observed each other, regardless of how they reacted. In some cases, a sloth bear was not aware of a nearby tiger but the tiger was aware of the bear. We recorded the date and time of the interaction, location, number of bears and tigers, presence of bear or tiger cubs, which species became aware of the other one first, how each animal responded, whether contact was made, and whether either species was injured or killed.

This study focused primarily on the behavior of bears after they became aware of a tiger. We categorized each interaction by the bear's initial behavior, which included the following: remain still, stand bipedally, charge, swat at, immediately fight, or flee. We recorded each species' response thereafter until the interaction ended by one or both leaving or one was killed. We classified interactions into three distance categories, relative to when the sloth bear first observed the tiger: <3 m (close encounters), 3–10 m (medium‐distance encounters), and >10 m (far encounters). We judged these distances using our perception of the sizes of the bears and tigers, and the angle from which the videos were shot (recognizing the potential for error in some cases).

We did not categorize distances if the video began after the bear had initially noticed the tiger, but we used these videos for other interpretations of how the interaction proceeded and ended. We also included cases where a tiger observed a sloth bear, but the sloth bear never noticed the tiger, because they are relevant to our hypothesis about why tigers can closely approach sloth bears (explained below). Finally, given that the videos did not always capture every moment of the interaction from beginning to end, we considered notes written by those who posted the video. Assessments of each video were conducted by each of three authors independently, and discrepancies rectified by further review and discussion.

## 4 RESULTS

We collected 40 videos and three photo‐sequences of interactions between sloth bears and tigers, posted during 2011–2023. Of the 43 total interactions, 37 involved a single bear and six involved a mother with one or two cubs; 37 involved a single tiger and six involved two or three tigers (two of these were a mother with cubs and four were multiple adults or subadults). In 32 cases, interactions were recorded from the beginning of the encounter, which we analyzed separately in three distance categories: 14 close encounters, 15 medium‐distance encounters, and three far encounters. In 19 of 25 interactions (76%) where it could be reasonably determined, tigers were aware of the bear's presence before the bear was aware of the tiger's presence. In two cases, the bear was clearly aware of the tiger's presence first, and in four interactions they appeared to notice each other simultaneously.

### 4.1 Close encounters

**FIGURE 2:**
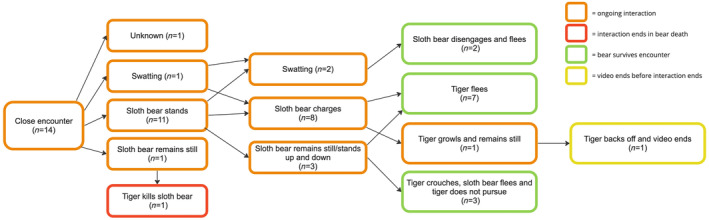
Interactions between sloth bears and tigers when the sloth bear first observed the tiger at close distance (<3 m), based on examination of video recordings posted online.

**FIGURE 3:**
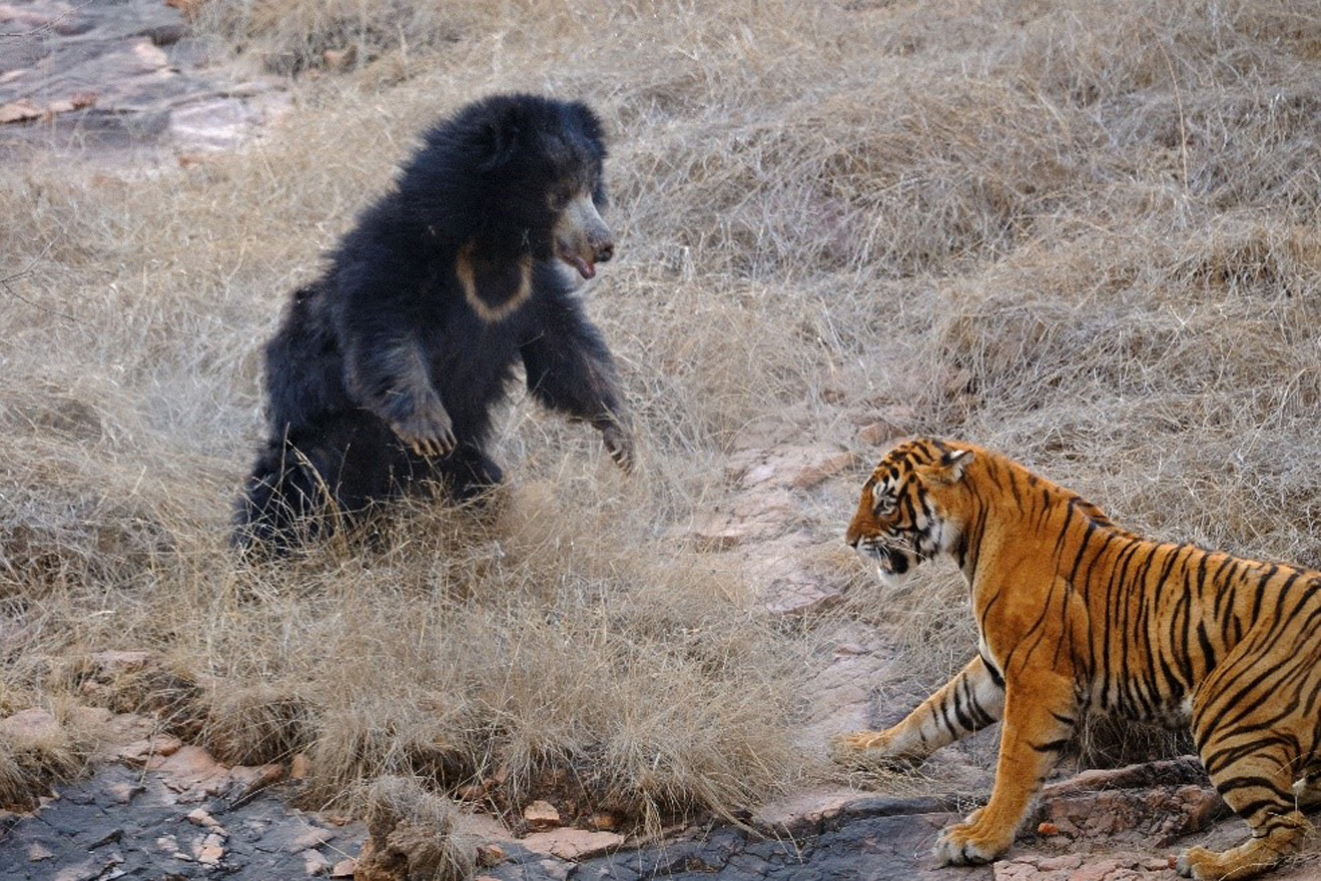
A typical posture of a standing sloth bear in reaction to a nearby tiger. This posture makes the bear look more intimidating, frees up the front claws as weapons, and enables it to lunge forward (Photo credit: Dicky Singh).

Of 14 encounters where the sloth bear first noticed the tiger when it was within 3 m (Figure 2), 10 (71%) were situations where the tiger was approaching the bear. In 11 of 13 (85%) encounters where it could be determined, the bear initially stood bipedally, and huffed (forcefully expelled air; Figure 3). In eight cases, the bear stood and then immediately charged (Table 1 and Video 1); all of these, except one, occurred when the bear first noticed a tiger that was actively approaching. In the other three cases, the bear was walking and happened upon a stationary tiger, at which point the bear stood but did not charge. In two of these cases, the tiger crouched and the bear ran off after a few seconds. In the other case, a mother with two cubs spotted a tiger behind a bush. All three family members stood up and down multiple times while the tiger watched but did not move.

**TABLE 1:** Initial reactions of sloth bears after observing a tiger at different distances.

Initial behaviors	Close: <3 m; *n* = 14 (%)	Medium: 3–10 m; *n* = 15 (%)	Long: >10 m; *n* = 3 (%)
Did not stand	14	53	100
Unclear if stood	7	40	0
Stood, did not immediately charge	21	7	0
Stood and immediately charged	57	0	0
Charged without standing	0	40	0
Swat at or struck	14	0	0
Remained still	7	40	100
Ran	0	20	0

*Note*: Table shows only the 32 cases where the initial reaction of the sloth bear was recorded on the video. Percentages in each column may total >100% because some categories are not mutually exclusive.

**VIDEO 1:** A video of a sloth bear standing, huffing and charging when surprised by a tiger (Video credit: Atik Ahmed).

In all eight cases, where the bear charged, it did not make contact with the tiger. In two other cases, the bear swatted at the tiger, and may have made some contact. One swatting match began when the bear was cornered by a tiger against a stone cliff wall. After a few seconds of the two animals swatting at each other, the bear found an opportunity to run off. In the second case, the bear was approached from the front while foraging, and suddenly saw the tiger when it was quite close, prompting it to stand and swat at the tiger.

**FIGURE 4:**
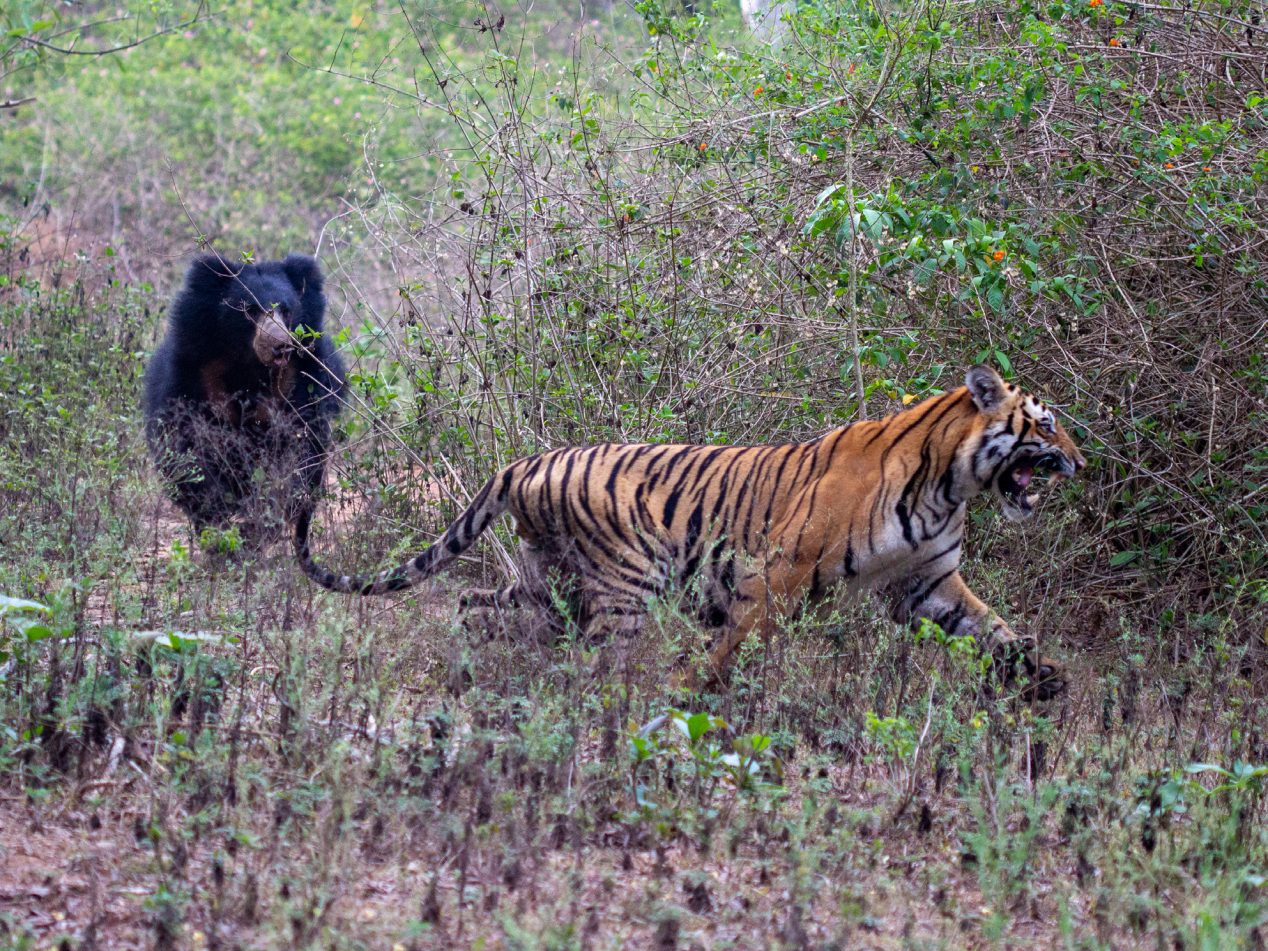
A sloth bear chasing off a tiger, but never attempting to catch it (Photo credit: Kabir).

In 13 of 14 close encounters, the bear either stood (readying itself for a fight), charged, or swatted at the tiger, and these ended with either the tiger (54%: Figure 4) or bear (38%) leaving, in each case unharmed. In the single case where a bear showed no aggression, but simply remained still and whined loudly, it was quickly dispatched, along with the cub on her back, by a large male tiger.

### 4.2 Medium‐distance encounters

**FIGURE 5:**
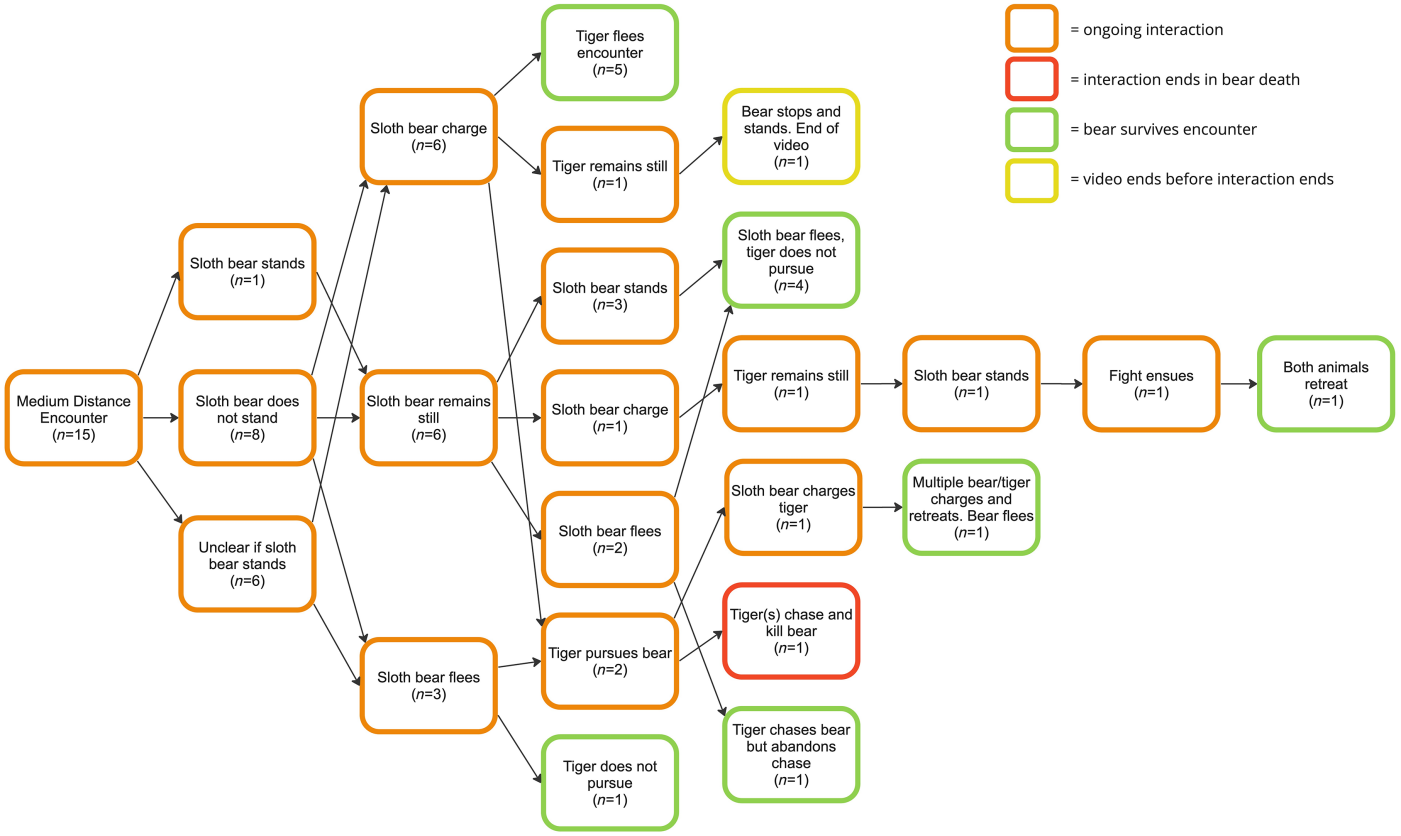
Interactions between sloth bears and tigers when the sloth bear first observed the tiger at a medium distance (3–10 m), based on examination of video recordings posted online.

Among 15 events where the tiger was first noticed at 3–10 m away (Figure 5), initial reactions of the bear were more variable than in close‐encounters, split between charging (40%), remaining still (40%), and fleeing (20%; Table 1). The bear initially stood up in just one of nine cases where we could tell with certainty. In the six encounters, where the bear initially remained still, apparently assessing the situation before reacting, its subsequent behavior was equally variable, from fleeing (33%), standing and then fleeing (50%), to charging the tiger (17%).

The incidents in which the bear spent time assessing the situation may be especially insightful in terms of understanding how it weighed its options, so here we look at these more closely. One such encounter involved a tigress with two cubs facing off with a sloth bear, 8‐m apart. The tigress seemed more interested in protecting her cubs than tangling with a sloth bear. After the bear stood several times, without the tiger reacting, it retreated into the forest and the tiger did not follow. In another case, a sloth bear came out of the forest onto a bluff overlooking a river and noticed a tiger resting in the water. The bear remained still and then began to walk backwards before turning and running back into the forest. The tiger initially followed, but returned to the river after being satisfied that the bear would not return. A third case involved a tiger lying in a dirt road with a sloth bear unknowingly approaching and noticing the tiger when about 5 m away. After remaining still several seconds, the bear stood up on its hind legs multiple times, with no reaction from the tiger. Eventually the tiger got up and slowly walked toward the bear, at which time the bear chose to run, checking back several times to ensure the tiger did not follow. In another similar case, a sloth bear was walking toward a waterhole; when it came around a large tree, it found itself face‐to‐face with a tiger lying in the water. The bear froze and the tiger did not move except to twitch its tail. After several seconds the bear made a sudden explosive run away from the tiger. After running 20–30 m, it stopped to look back and found it was not being pursued. Consistent among these cases in which the bear fled is that it met the tiger incidentally (i.e., was not being stalked), and in three cases the tiger was content resting on a road or in a waterhole, and the bear had time to leave the scene.

The single incident in which the bear initially remained still, but then decided to charge involved a large male tiger. The video began with the bear watching a tiger cross ~7 m in front of it, but not coming toward it. Instead of waiting to see whether the tiger would pass by, the bear charged, possibly agitated by the tiger's large size, and the two animals exchanged swatting, without making significant contact. The video ended with both animals backing away.

Two encounters documented a tiger stalking a sloth bear cub, each about 1 year old (too old to be riding on the mother's back). One video began with a tiger watching a sloth bear family that was unaware of the tiger. The tiger slowly stalked one of the cubs, which had wandered ~5 m from its mother. Suddenly, the tiger stopped stalking and turned in the direction of the mother bear, who was charging, causing the tiger to flee. Another similar encounter showed a tiger on a small rise looking down on a sloth bear family. When ~5 m away, the tiger sprinted toward one of the cubs. The cub tried to run, but the tiger tackled it. The mother bear, as well as the cub's sibling, charged the tiger, attacking it in the long grass until the tiger, overwhelmed by the onslaught, ran off with all three bears in pursuit. The cub survived but with an apparent injury to its right rear leg.

Of 14 medium‐distance encounters where the final outcome was filmed, seven ended when the bear fled and was not pursued, five when the tiger fled, one when both retreated, and one where the bear was killed by three tigresses after it tried to run. Two bears were noticeably injured (the cub with an injured leg, and a mother with a cut on her chest).

### 4.3 Far encounters

We found only three cases where the sloth bear first noticed a tiger that was >10 m away, probably because people were less likely to video and post cases where the two animals were far apart, and no interaction occurred. The small sample also may indicate that sloth bears were often unaware of potential threats at this distance (discussed more below). In one case, the bear noticed the tiger some distance behind it, and continued to walk away in a leisurely fashion, occasionally looking back to observe the tiger. At one point, the bear almost came to a standing position, but did not when it noticed that the tiger had stopped moving and was crouching. It walked into the brush and the tiger did not pursue. In two cases the bear initially remained still when it first saw the tiger. In one incident, the tiger got out of a waterhole and approached the bear in clear view. In response, the bear moved toward the tiger and eventually stood and charged, chasing the tiger away from the waterhole. In the other case, the bear and tiger watched one another as the bear stood at the edge of the woods and the tiger sat on a concrete feature near a dirt road. When the tiger did not approach, the bear altered its course and moved around the tiger, staying in dense vegetation. The tiger paid close attention to the bear's movements as it walked around it, but did not leave the concrete feature nor move toward the bear. By the end of the video the bear had moved on its way and the tiger seemed at ease.

### 4.4 Unknown initial distances

We examined nine events in which the video began after the bear first noticed the tiger. One showed a tiger chasing a bear, and another was a bear chasing a tiger. Two encounters included multiple charges by a bear attempting to chase off a tiger, but the tiger, after initially backing off from the charge, continued to pursue the bear. In one of these encounters the tiger eventually stopped pursuing the bear and allowed the bear to run off. In the second, which took place at night, the end of the interaction was not caught on video, but the accompanying notes indicated that the tiger eventually killed the bear. Another encounter showed a tiger approaching a sloth bear that looked to be injured. According to notes and testimonials about the interaction, the tiger had been hunting the bear and ultimately killed it.

Several of the videos that started after the initiation of the interaction were rather unusual. One began with a tiger approaching a sloth bear in a tree. We do not know whether the bear climbed the tree to escape the tiger, or was there for another reason. The tiger climbed partway up the tree, but did not reach the bear; the bear did not climb higher, but clumsily adjusted itself in a crotch and faced the tiger, which eventually retreated without contact. Another began with a sloth bear slowly backing off from two tigers (a mother and large cub). It turned and sprinted about 40 m away from the tigers before turning back briefly to ensure that they were not following. Another video showed a sloth bear chasing off young tigers. According to the notes the tigers were playing with the bear and not a serious threat. Finally, one photo‐sequence showed a small sloth bear charging a satiated large male tiger, which was lying down. The bear ran off and the tiger did not stir.

### 4.5 Unobserved tigers

We examined two videoed events where a sloth bear never became aware of a tiger that was within 10 m and watching the bear intently. One bear walked 7–10 m in front of the tiger, which never moved. The other bear walked toward a river, while being watched by a tiger on a rise along the river bank. The tiger made several starts toward the bear, but eventually just crouched and watched. The bear finished drinking and walked into the woods, apparently unaware of the tiger.

### 4.6 Summary of all encounters

Bears stood on their hind legs in 24 (56%) of the 43 interactions, and charged the tiger in 24 interactions (not necessarily the same ones). Bears nearly always stood up immediately upon seeing a tiger within 3 m, but seldom stood when spotting a tiger farther away (Table 1). The one individual that did not stand when the tiger was at close range was immediately killed.

The vast majority (*n* = 37, 86%) of incidents ended without any, or very little, physical contact. None resulted in significant injury or death to a tiger. Two sloth bears were noticeably injured, and two videos captured the death of a sloth bear; additionally, according to notes, two other sloth bears died off camera, for a total of four fatal interactions (9.4%) and five bears (one mother and cub) killed. Three of these fatalities were caused by a large male tiger, and one by three young tigresses.

The length of the interaction was generally determined by the tiger, as in most interactions the bear was looking to escape. Interactions ended with either the tiger being chased off, the bear running or walking off and the tiger not pursuing, or the bear being killed. The longest fight lasted roughly 15 min, and occurred between a large male tiger and a female sloth bear who had a yearling offspring alongside. Several times the tiger's jaws were gripped on the mother's neck (Video 2). She eventually escaped with evident injuries. Although the tiger was not noticeably injured, it appeared physically exhausted.

**VIDEO 2:** Video of a female sloth bear, with yearling, surviving an extended fight with a tiger, with the ruff of hair around her neck protectng against the tiger's grip (Video credit: Akshay Kumar).

## 5 DISCUSSION

### 5.1 Patterns in responses of sloth bears to tigers

Aside from humans, tigers are the only significant predator on bears in Asia. Leopards (*Panthera pardus*), as well as pack hunters such as wolves (*Canis lupus*) and dholes (*Cuon alpinus*) may be capable of killing bear cubs and possibly juveniles, but they pose little threat to adult bears. In Southeast Asia, sun bears (*Helarctos malayanus*) and Asiatic black bears (*Ursus thibetanus*) occur regularly in tiger scats (Kawanishi & Sunquist, 2004; Naing et al., 2020; Vongkhamheng, 2011). In a hilly evergreen forest of Laos, Asiatic black bears accounted for 10% of the tiger diet in terms of ingested biomass (Rasphone et al., 2022). In the Russian Far East, Asiatic black bears or brown bears occurred in 8.4% of Amur tiger scats, and represented 2.2% of tiger kills (4.5% during the non‐hibernating season); predation was mainly on adult bears (Seryodkin et al., 2018). Kerley et al. (2015) indicated that whereas bears are far less important than ungulates in the diet of Amur tigers, they still represent a significant dietary component, comprising 4–13% of ingested biomass. In India and Nepal, sloth bears can make up ~2% of a tiger's diet (Andheria et al., 2007; Biswas & Sankar, 2002; Kapfer et al., 2011; Ramesh, Snehalatha et al., 2009; Reddy et al., 2004; Sankar & Johnsingh, 2002). However, there are also many places where significant populations of sloth bears and tigers overlap, but sloth bears have not been detected in the tigers' diet (e.g., Bhandari et al., 2017; Biswas et al., 2023; Hayward et al., 2012; Pun et al., 2022).

Sloth bears were injured in two cases and killed in four of 43 encounters that we observed. However, the vast majority of encounters involved no contact, and no injury to either species. Likewise, Joshi et al. (1999) witnessed four encounters between sloth bears and tigers in Nepal, each of which involved aggressiveness by the bears, and all ended without injury to either species. Thus, it appears that the aggressive intimidation that sloth bears use to defend themselves from tigers, involving standing, charging, vocalizing, and occasionally slapping at a threatening tiger, is typically a successful deterrent. Sloth bears employ a vigorous offense as a defense against a superior predator. This strategy sometimes fails against a large male tiger or groups of tigers, but is still more effective than running. One of the cases described by Joshi et al. (1999) involved a female sloth bear with cubs successfully fending off three tigers.

**FIGURE 6:**
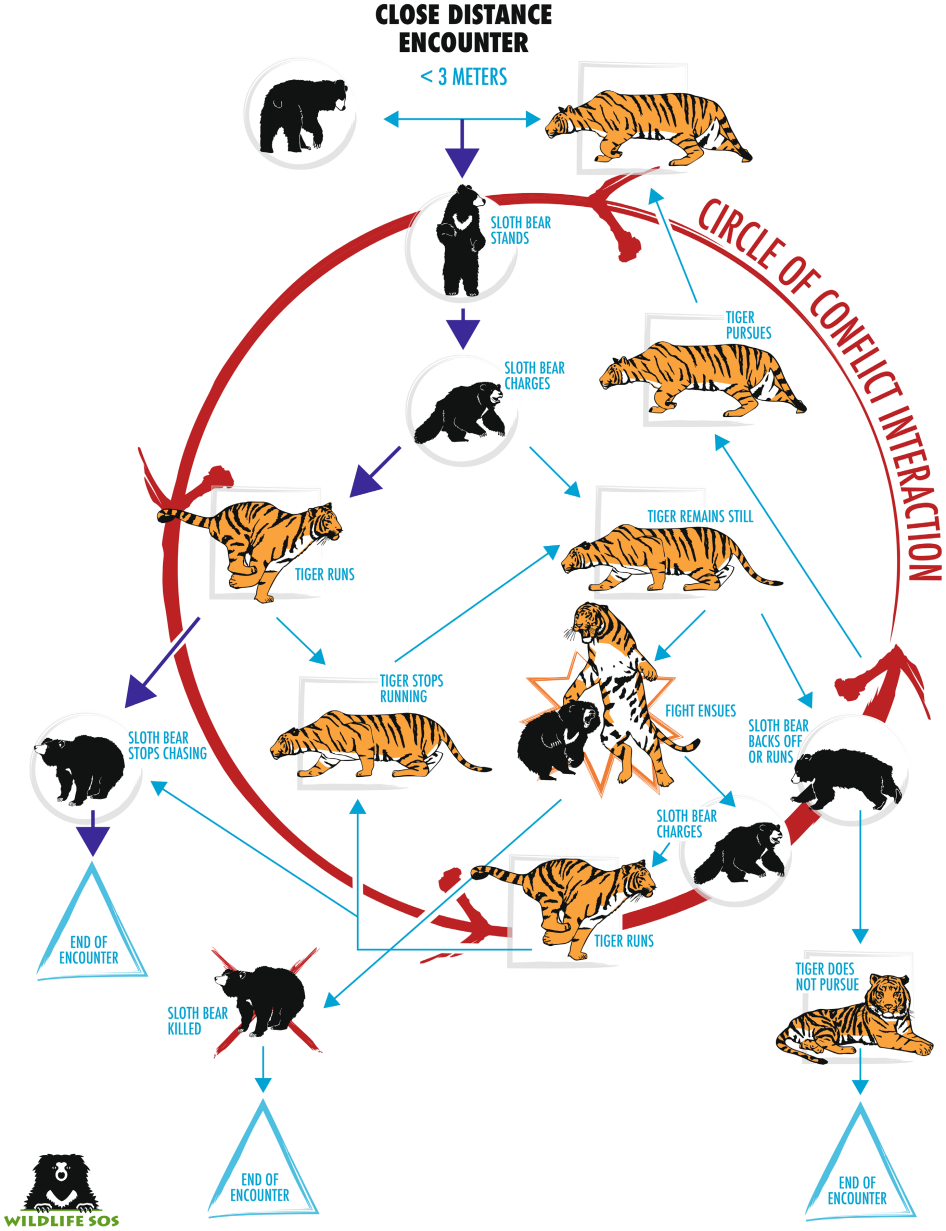
General patterns of interactions when sloth bears encounter tigers at close range (<3 m).

Close encounters between sloth bears and tigers generally followed a predictable sequence of behaviors (Figure 6). When first noticing a tiger within 3 m, the bear almost always reacted explosively, standing and then charging. Depending on the tiger's reaction, the bear might repeat this several times; hence, we call this the “circle of conflict interaction.” However, most interactions ended after a single charge by the bear, indicating the general success of this strategy.

**FIGURE 7:**
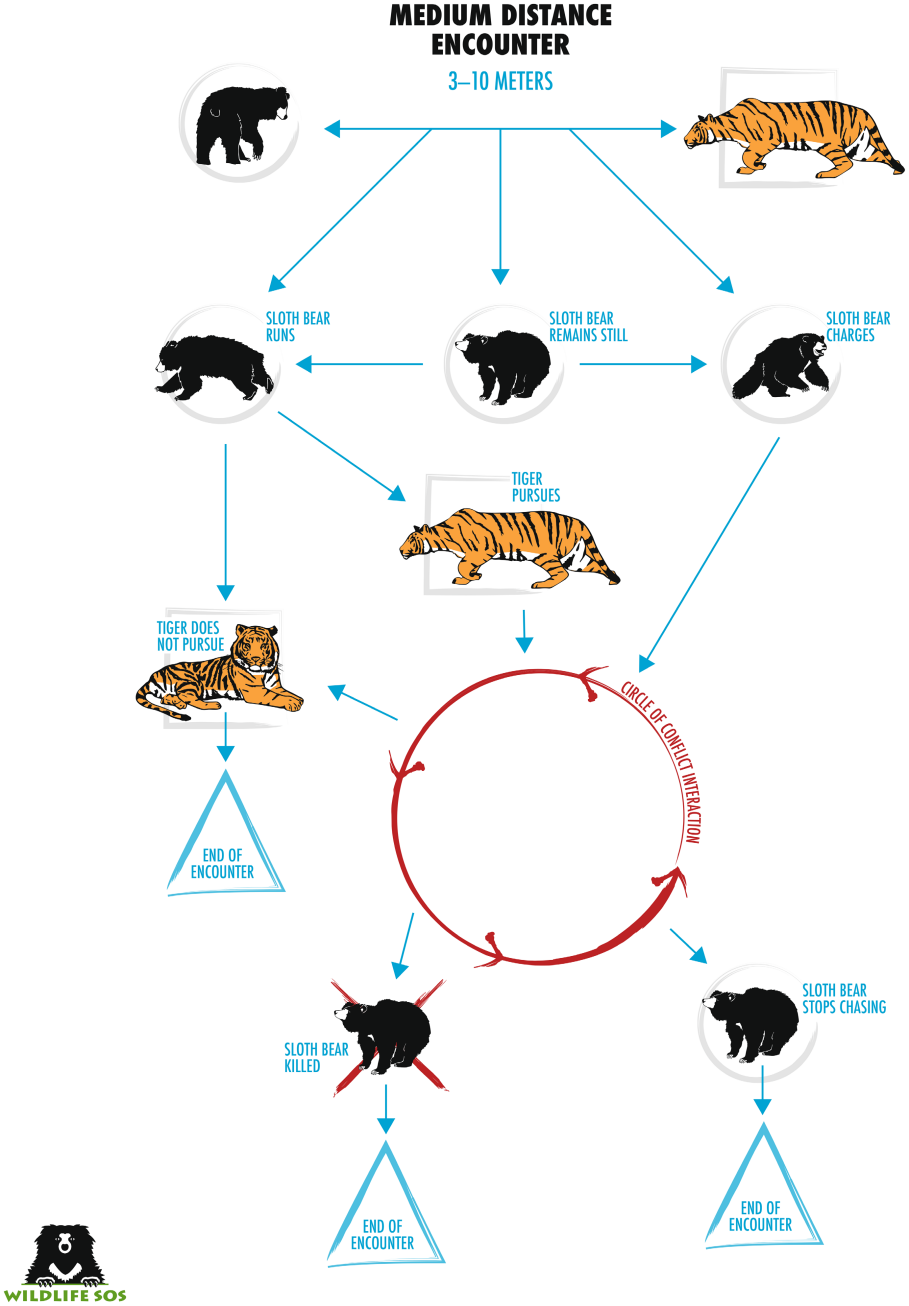
General patterns of interactions when sloth bears encounter tigers at medium range (3–10 m).

When sloth bears first noticed a tiger 3–10 m away, their initial response was more varied (Figure 7). Some bears charged and followed the “circle of conflict interaction,” whereas others either ran away or remained still to assess the situation further. We could not tell what motivated these different reactions, but we suspect decisions were influenced by the relative sizes of the bear and tiger (and number of tigers), behavior of the tiger, obstructions between the bear and tiger, availability of an escape route, and possibly the bear's past experiences with different strategies.

Bears that noticed tigers >10 m away did not necessarily need to make a fight‐or‐flight decision, but had time to observe the tiger's behavior. One long distance encounter prompted a charge, possibly motivated by the bear's desire to make use of a waterhole, and the tiger's aggressive movement toward the bear.

These patterns indicate that sloth bears are not hard‐wired to charge, but employ that strategy when the threat level is high. Charging puts the bear closer to the tiger, where the risk of contact and injury is greater. But the strategy seemed to work in intimidating tigers and mainly avoiding physical contact. Bears commonly stood up when faced with a nearby threat, but typically did not immediately stand when the tiger was >3 m away, indicating that the bipedal posture was not just intended to get a better look at the tiger. Standing up likely serves to make the bear look larger, free up its front claws as weapons, and provide initial forward momentum when it drops to its front feet and lunges.

Some bears continued to chase even after a tiger fled, helping to ensure that the tiger would not return. In interpreting this behavior, it is important to recall that sloth bears do not prey on large vertebrates as food, so their aim is not to kill the tiger, but to defend themselves and prevent being predated upon. If a bear can significantly increase the tiger's perception of risk, that may dissuade the tiger from attacking. Tigers and bears may perceive the risks of fighting differently: a tiger that sustains a physical injury in a fight with a bear may inhibit its ability to prey on larger mammals, whereas non‐life‐threatening injuries are less likely to impact a sloth bear's feeding. Although our study did not focus on tiger behavior, we found it noteworthy that tigers eventually retreated from 42% (*n* = 18) of the encounters while sloth bears were killed or retreated in 37% (*n* = 16).

The risk of a bear killing a tiger may be small, but not zero. In 2019, a tiger was found dead by a waterhole in Dudwa National Park in the Indian terai. Post‐mortem examination suggested that the tiger's injuries were inflicted by a sloth bear. Camera trap images revealed a large sloth bear leaving the area by the waterhole near the time of the tiger's death. The bear was clearly injured and had scratches on its face (Singh, 2019).

### 5.2 The role of myrmecophagy

In all areas within the geographic range of sloth bears, termites or ants are a dietary staple during some portion of the year (Baskaran et al., 2015; Garshelis et al., 1999; Joshi et al., 1997; Khanal & Thapa, 2014; Laurie & Seidensticker, 1977; Philip et al., 2021; Ramesh, Sankar, & Qureshi 2009; Rather et al., 2020). Whereas sloth bears are an obligate myrmecophage, they also consume a variety of fruits, when available. Their geographic range on the Indian subcontinent has generally low fruit abundance but a high density of termites, conditions not suitable for other species of bears (Steinmetz et al., 2021).

Other bear species also consume insects, and the sun bear in particular has a very long tongue adapted for feeding on stingless bees (Fredriksson et al., 2006). However, among all eight bear species, the sloth bear's adaptations for insect feeding are most pronounced. They are missing two front upper incisors, have large protrusible lips and an extended broad palate (all for sucking), nostrils that they can voluntarily close (to prevent inhalation of insects), a long shaggy coat (thought to be a defense against biting ants), and long, slightly curved, front claws with in‐turned feet (adapted for digging).

Some physical features are also adaptive in interactions with tigers. Their long front claws are formidable weapons, as are their large canines, which are similar in size and strength to those of carnivorous polar bears (*U. maritimus*) and brown bears (Christiansen, 2008). Their long, shaggy coat not only protects them from insect bites, but also the bites of tigers. The very long ruff of hair around the neck seemed to protect the bear whose neck was in the jaws of a tiger (Video 2). The prominent white chest marking may help the bear appear larger and more intimidating: when sloth bears stood, they spread their front legs, drawing attention to this marking (Figure 3).

Whereas sloth bear claws are well‐adapted for excavating termite colonies, they are blunt and not good for climbing. This restriction sets them apart from Asiatic black bears and sun bears, which also historically existed with tigers; whereas Asiatic black bears and sun bears regularly forage in trees, and use trees as a refuge, sloth bears infrequently use trees. Even sloth bear cubs do not use trees as a refuge, but instead are carried on the back of their mother for 6–9 months. Among ursids, this behavior is unique to sloth bears, but is common in other myrmecophagous mammals. Joshi et al. (1999) reasoned that cub‐carrying by sloth bears was not an adaptation to myrmecophagy, per se, but rather a defense against tigers by a species that (due to its myrmecophagy) could not rely on tree‐climbing for escape.

**FIGURE 8:**
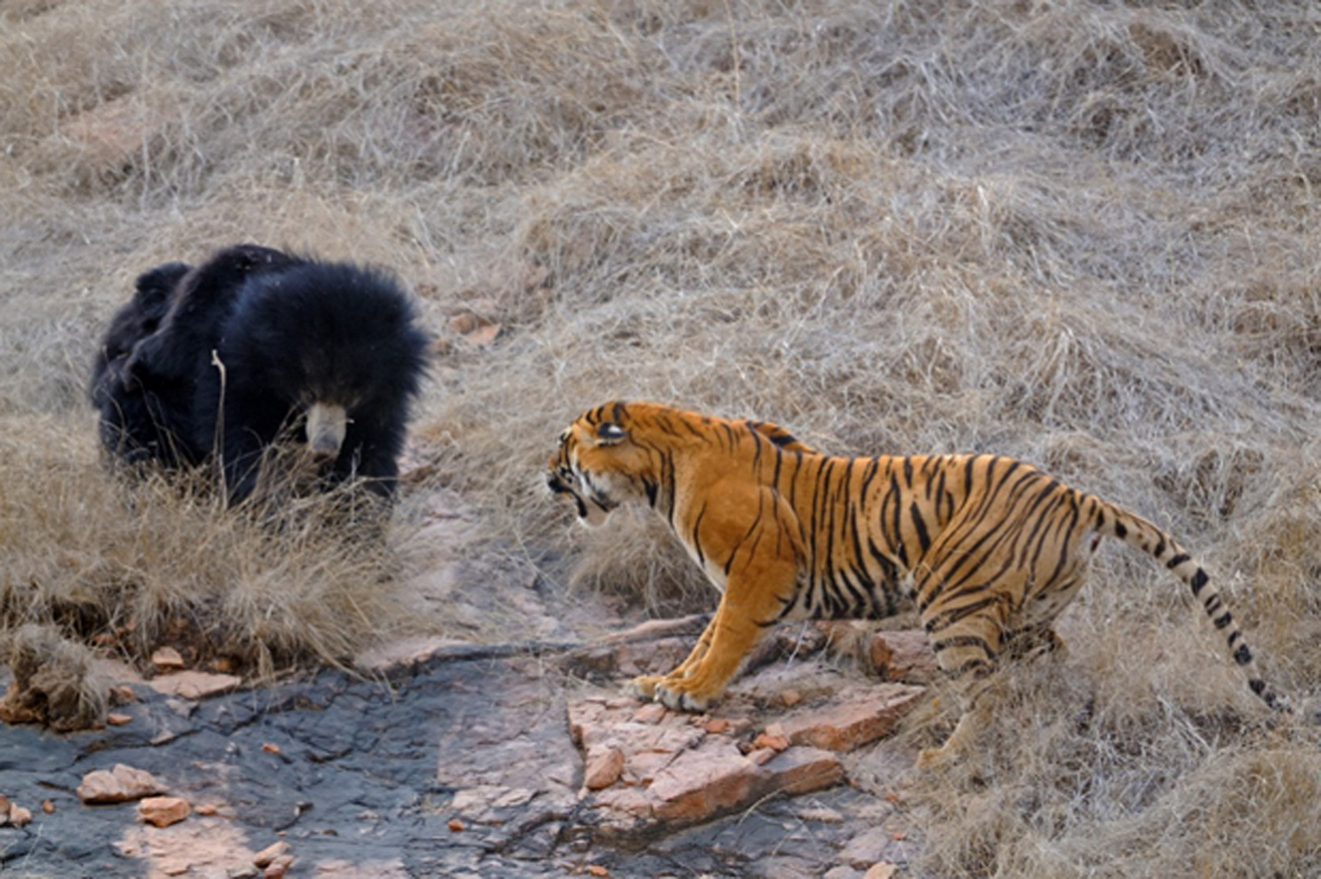
A sloth bear mother carrying cubs on her back during an interaction with a tiger (photo credit: Dicky Singh).

The sloth bear mother's long, shaggy coat not only serves as protection against both insects and tigers, but also provides a surface for the cubs to cling to. Sloth bears carry cubs while traveling, digging termite mounds (Joshi et al., 1999), and even during aggressive conflicts with a tiger (Singh, 2011; this study, Figure 8). On their mother's back, the cubs are safer, and the mother has the advantage of knowing where they are during a frenetic tiger encounter. Seryodkin et al. (2018) suggested that Amur tigers may take advantage of the vulnerability of brown or Asiatic black bear mothers while protecting cubs on the ground.

Sloth bears carry their head quite low while slowly walking and focusing on underground scents (Laurie & Seidensticker, 1977), enabling them to detect termite colonies 50–80 cm (up to 1.5 m) underground (D. Garshelis, unpublished observations, 1990–1993), but their attention to the ground detracts from their awareness of tigers. Also, their foraging involves blowing away soil and sucking up termites and ants (Joshi et al., 1997), creating significant noise, thus attracting attention of tigers as well as making it difficult for them to hear an approaching tiger. These foraging behaviors interfere with the bear's ability to be vigilant, and as a result, tigers are able to approach quite close, even in open habitat, before being detected. One video documented a tiger touching the hindquarters of the bear, at which point the bear whirled around, stood up, and charged (Video 1).

### 5.3 Evolving with large predators

The sloth bear evolved on the Indian subcontinent, and fossil evidence, though generally poor (Advait Jukar, Yale Institute for Biospheric Studies, personal communication, 2021), suggests that they existed in their current form since the early Pleistocene (1.8–0.6 million years ago; Lydekker, 1886). Genetic studies indicate that sloth bears are much older (4–6 million years; Kumar et al., 2017; Nyakatura & Bininda‐Emonds, 2012; Yu et al., 2007; Zou et al., 2022). Erdbrink (1953) presented good evidence that this species never ranged beyond present‐day India, Sri Lanka, the narrow terai of Nepal, and Bangladesh (where they are now extirpated: Islam et al., 2013).

Tiger lineage likely began in north‐central China (Lou et al., 2019; Mazak et al., 2011), with fossils dating back to the basal Pliocene, ca. 2 million years ago (Lou et al., 2019). However, tigers did not cross the Himalayas and enter the range of sloth bears until the late Pleistocene (20,000–52,000 years ago), then spread across India 12,000–16,000 years ago (Cooper et al., 2016; Kitchener & Dugmore, 2000; Luo et al., 2019). Thus, sloth bear behavior has been shaped by the presence of tigers for at least 20,000 years.

**FIGURE 9:**
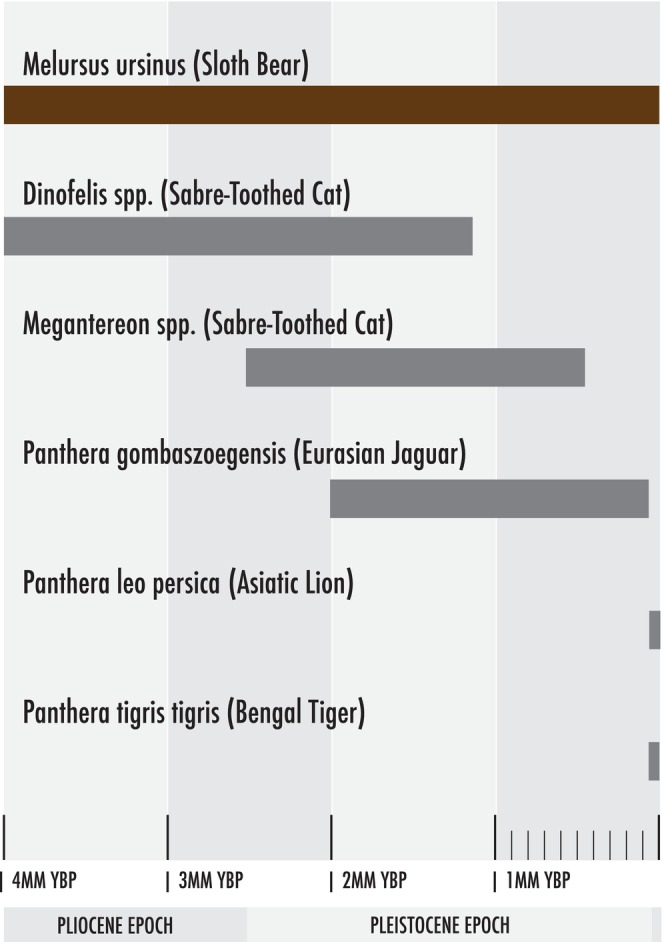
Stratigraphic ranges of sloth bears and large cats that were likely predators of sloth bears.

**FIGURE 10:**
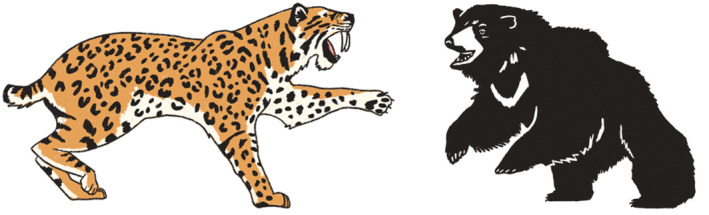
Conceptual interaction between *Megantereon* sp. (an extinct large cat) and a sloth bear (drawing: Kara Mohr).

Other large predators existed well before the arrival of tigers on the Indian subcontinent, which also likely affected the evolution of sloth bear behavior. The concept of a species having traits that link back to a long extinct predator (ghosts of predators past) is not new. The pronghorn (*Antilocapra americana*), the fastest land mammal in North America, apparently developed its speed in reaction to long extinct predators such as the North American false cheetahs (*Miracinonyx inexpectus* and *M. trumani*) and long‐legged hyaenas (*Chasmaporthetes* sp.; Byers, 1997). Sloth bears overlapped with many large felid predators, including *Dinofelis, Megantereon* and *Panthera* (Figure 9). *Dinofelis* was a saber‐toothed cat that lived 5–1.2 million years ago (Jiangzuo & Liu, 2020; Werdelin & Lewis, 2001). *Dinofelis cristata*, the likely species that sloth bears would have encountered, is thought to have behaved and hunted like a lion or tiger (Anton, 2013). *Megantereon* was another saber‐toothed cat (Palmqvist et al., 2007), that overlapped with sloth bears from roughly 2.5–0.5 million years before present (Figure 10). The fossil record suggests that *Megantereon* found on the Indian subcontinent (*M. whitei or M. falconeri*) were the largest of the genus, roughly reaching tiger size, though more heavily built like a jaguar (*Panthera onca*, Matthew, 1929; Navarro & Palmqvist, 1995; Palmqvist et al., 2007; Pilgrim, 1932). The Eurasian jaguar (*Panthera gombaszoegensis*) may have overlapped with sloth bears from 2 million until about 35,000 years ago (Jiangzuo & Liu, 2020). Finally, the Persian lion (*Panthera leo persica*) overlapped with sloth bears in western India during the late Pleistocene (Barnett et al., 2014; Divyabhanusinh, 2014; Pocock, 1930; Schnitzler & Hermann, 2019). This host of large felids certainly posed a significant threat to sloth bears, which helped develop their defensively‐aggressive behavior long before they began interacting with tigers.

### 5.4 Implications for sloth bear attacks on people

Sloth bear attacks on people are a major conservation issue for the species (Dharaiya et al., 2020). Results of this study lead us to believe that sloth bear aggression toward tigers is a pattern of behavior that extends to encounters with humans. Our improved understanding of what provokes their explosive attacks against tigers may therefore help in reducing attacks on people. Notably, the initial behaviors of a sloth bear to a tiger at close quarters (standing, huffing, and charging), are also commonly reported by people who have been attacked by sloth bears (Bargali et al., 2005; Ratnayeke et al., 2014). Bear species across the globe largely view humans as a potential threat, but whereas most bears flee at the earliest opportunity, or possibly bluff charge (not making contact), sloth bears are apt to charge and swat at a person's face. This behavior is well known to local people, justifying their common fear of this species.

Because sloth bears tend not to be vigilant, an approaching tiger or person can often get quite close without being noticed. That would work in favor of a tiger attempting to prey on a bear, but would create a dangerous situation in the case of a person unwittingly approaching an unwary bear. It is not surprising that the majority of sloth bear attacks are due to surprise encounters at close range (Ratnayeke et al., 2014; Sharp et al., 2020; Singh et al., 2018). Hence, the first obvious recommendation for people living or working in habitats occupied by sloth bears is to try to avoid close‐range encounters by making noise (Bargali et al., 2005; Debata et al., 2016; Dhamorikar et al., 2017; Garcia et al., 2016; Ratnayeke et al., 2014; Sharp et al., 2020; Singh et al., 2018). However, if a person does encounter a bear at close range, and the bear did not yet notice, then it is best to back off quietly, so as not to draw the bear's attention. The ideal outcome is for the bear to continue what it was doing, never aware of the person.

If a sloth bear does notice a person at close quarters, and if they respond to the human threat as they would to a tiger, then the bear is likely to charge. Fighting an attacking bear may lead to the bear fighting back harder, just as they do with tigers. Falling to the ground, covering up, and playing dead, as suggested by several authors (Ratnayeke et al., 2014; Sharp et al., 2020), is a good way of diffusing the situation, enabling the bear to feel less threatened and leave, while at the same time putting the person in a protective position. This recommendation is the same for defensive attacks by brown (grizzly) bears and Asiatic black bears (Herrero, 2002; Mir et al., 2023); however, unlike these other bear species, which might be intent on consuming a person—in which case playing dead is ineffective—sloth bear attacks are only intended to deter the intruder from attacking.

Tigers, of course, never play dead, but rather run away. Here the tiger response and recommended human response diverge, for several reasons. In our study, of the 18 cases where tigers fled, bears pursued them, at least a short distance, 61% (*n* = 11) of the time. The bears' intent was not to catch the tiger, but to ensure that it was well clear of the area and no longer a threat. Tigers can run faster than bears, so the bear is unlikely to catch a tiger running away, unless the tiger decides to stop and fight the bear. If a sloth bear reacts similarly to a person who runs, they are likely to catch the person; this may mirror the case of a tiger changing its mind and deciding to fight. The bear is unlikely to discern that the person is a not continuing threat. For this reason, it is unwise to run from a sloth when encountered at close range: many people have been injured or killed attempting to do so (Sharp et al., 2020).

Sloth bears may alter their response strategy depending on their perception of risk. Charging multiple tigers is more apt to have a poor outcome, so in the few such cases that we observed, sloth bears opted to run or back off rather than charge. Likewise, whereas a sloth bear may feel confident in attacking a single person, a group of people is likely to be more intimidating, and reduce the chance of a bear attacking.

In today's age, most people are not actively hunting sloth bears, and do not pose a threat akin to a tiger, but the bears do not know that. Bouskila and Blumstein (1992) wrote that animals rarely have perfect information, and generally are expected to maximize fitness by overestimating rather than underestimating risk. Overestimation costs, such as expended energy, have milder fitness consequences than underestimating the danger, which might result in immediate death. The extreme reaction of a sloth bear to a human walking by may often be a case of overestimating risk.

Explosively charging and attacking a potential threat has served sloth bears well for hundreds of thousands, if not millions, of years. Only in recent times has this defensively aggressive behavior become an issue for the conservation of the species: sloth bears that attack people are often killed, and local people that fear these bears are often not inclined to favor the presence of nearby populations. Indeed, people's adverse reactions to this species represents one of the largest obstacles to the conservation of this species, and one of the paramount reasons that it remains Vulnerable on IUCN Red List (Dharaiya et al., 2020).

### 5.5 Comments about the data

The data used in this study were derived from people taking videos or photos and posting them to the internet or social media. This form of crowdsourcing, where the data collection is not organized, directed, overseen, or even solicited by any scientist, has been called “passive citizen science” (Edwards et al., 2021; Ghermandi & Sinclair, 2019). Those who contribute such photos do not intend them to be used for any scientific purpose, and may never know that they were. Notably, citizen science already is subject to issues of quality control, and this unguided and unintended data collection has even fewer assurances of data reliability; nonetheless it is a valuable source of certain kinds of information that are otherwise difficult to obtain.

Jarić et al. (2020) reviewed the burgeoning use of online photos and other data in ecological research, what they called “iEcology.” Posted videos and photos have been exploited as data in situations where formal studies are particularly difficult (Coram et al., 2021; Morais et al., 2021; Węgrzyn et al., 2023), and may be used to study behavior (Hernandez et al., 2019; Jagiello et al., 2019), as we did. Authors have commented that, due to the happenstance and human‐filtered nature of the data that are posted, researchers need to be wary of potential biases. Conversely, a major strength of passive citizen science is that because it employs photos taken opportunistically, it can capture rare events that would likely be missed in a more structured data collection effort. Those who posted the videos that we used were generally tourists, naturalists, or professional photographers on safaris, who hoped to see and film wildlife, but just happened to observe a sloth bear interacting with a tiger. It is likely that many of the photographers centered their observations at water sources, where these animals are more apt to be seen, and also more likely to interact (19% of our cases were at water sources).

Whereas a bias may be introduced both in terms of what people tended to record, and also what they chose to post, this bias actually promotes the documentation of unusually‐observed events, such as the interactions between sloth bears and tigers. The people who captured sloth bear–tiger interactions would be most likely to record and post the most exciting events, such as a charging bear or fight between a bear and tiger. However, just seeing a bear and tiger in close proximity is quite rare, and not knowing in advance what will occur, it seems reasonable to assume that most people would attempt to photograph the ensuing events. Dense vegetation would make recording more difficult (and yield photos that are less apt to be posted), but it is not obvious that this introduces a bias insofar as how the bear reacted to the tiger. It is possible that bears feel less threatened and more able to sneak away in dense cover, but it is also possible that dense cover is favorable to the tiger's attempted predation on the bear.

Encounters between tigers and bears may unfold somewhat differently during darkness, a time when tigers often hunt (Karanth et al., 2017), and when bears may be at a greater disadvantage of being ambushed. It is notable that whereas other bear species tend to be diurnal and crepuscular (except when in proximity to humans; see review by Paisley & Garshelis, 2006), sloth bears are generally more active at night, probably to avoid daytime heat (Bargali et al., 2012; Joshi et al., 1999; Ramesh et al., 2013; Yoganand et al., 2013). Being active at night may increase their ability to rapidly respond to a tiger, or conversely may increase the probability of attracting the attention of a nocturnally hunting tiger. In Nepal, Joshi et al. (1999) found that juvenile sloth bears and females with cubs, the classes least able to defend against a tiger, differed from other bears in being diurnally active. Although we examined just one nighttime video (where accompanying notes indicated that the bear was killed), our sample of videos were recorded at various times from morning till evening, and the bears involved appeared to be well balanced in terms of sex, age, and family groups.

We believe that the largest potential bias in our data is that people were more likely to post events that would appeal to an audience, particularly with lots of action, as opposed to a bear and tiger that saw each other and just sauntered away. As such, the dataset may over‐represent the proportion of encounters that prompt the bear to charge the tiger. Whereas this is something to be aware of in datasets of this nature, we do not think it alters the general conclusion that sloth bears often react to tigers with vigorous defensive aggression. Moreover, as we have shown, this makes sense in terms of their evolution and natural history, fits with how they commonly react to people, and appears to be very effective against tigers.

The biggest constraint that we encountered in extracting data from these videos is that often they represented just a portion of the event, either because the person did not witness the start of the interaction, or because only a part of the video was posted. Thus, we could not always tell when the bear actually first detected the tiger. In one case, a bear appeared injured when the video began, but we did not know if the injury occurred during that encounter or sometime previously.

A notable aspect of the videos that we examined is that, for the most part, the bears and tigers ignored the human observers, apparently being habituated to frequent tourists in vehicles. Often the engines were started to move the vehicle to provide a better view. Human voices were commonly heard, as people reacted to the events. Periodically the bear or tiger would look at the human observers, but only in a few cases did human disturbance appear to affect the outcome (e.g., one of the animals leaving the scene).

Datasets like this one, derived from non‐scientists taking photos and posting them on social media or websites, are likely to become increasingly common, and may be a new avenue for exploring animal behavior. Just 5 years ago this study would not have been possible, and in fact, from the time that we conceived this study and collected the initial batch of available videos, our sample size dramatically grew, suggesting that over the next decade, much more intriguing information and new insights will become available.

## AUTHOR CONTRIBUTIONS

**Thomas R. Sharp:** Conceptualization (equal); data curation (equal); formal analysis (equal); investigation (equal); methodology (equal); visualization (equal); writing – original draft (equal); writing – review and editing (equal). **David L. Garshelis:** Conceptualization (equal); investigation (equal); methodology (equal); writing – original draft (equal); writing – review and editing (equal). **Wesley Larson:** Data curation (equal); investigation (equal); methodology (equal); visualization (equal); writing – review and editing (equal).

## CONFLICT OF INTEREST STATEMENT

The authors declare no conflicts of interest.

## Data Availability

All data are available as tables in the Appendix Tables A1 and A2.

## APPENDIX 1

**TABLE A1:** Sloth bear tiger interactions, locations and short narratives.

#	Location	Narrative
1	Ranthambore N.P.	[Video] A tiger was stalking a sloth bear, and the bear was unaware of the tiger. The bear finally noticed the tiger right behind it and immediately stood up and huffed. It then jumped up and charged the tiger chasing it while the tiger jogged away from the bear. The bear chased the tiger about 20 m until they approached the second tiger, which was crouched down on its haunches. The bear did not notice a second tiger until it stood up. The bear reacted by standing up on its two hind legs and huffing again. The bear then chased the second tiger a few meters. The bear then stood up a few more times, but the tiger remained still. The bear then turned and ran about 20 m away from the tigers before stopping to look back at the tigers, which had not moved. The sloth bear continued to walk away with both tigers looking on, but not pursuing
2	Ranthambore N.P.	[Photographs and Narrative] A female sloth bear stumbled upon two courting tigers. The female tiger noticed the sloth bear and went over to investigate. By the time the mother bear was aware of the tigress, she was very close. The sloth bear charged the tigress, who backed up, but did not run at first. The bear stood up on two legs and began making loud noises. The two traded paw swipes, with the bear getting the better of the integration. The tigress hastily retreated. The large 4‐year‐old male tiger then approached the bear. These two swatted at each other, and the bear again got the better of the exchange. The male tiger quickly retreated. The mother bear hastily walked off
3	Ranthambore N.P.	[Video] The tiger was crouched, watching the bear from roughly 5 m away. Eventually, the tiger crept up behind the sloth bear and swatted the bear's left haunch with its left paw. The bear spun to its left and stood while huffing, and then huffed a second time in the tiger's face and quickly charged the tiger. The tiger quickly retreated with the bear huffing and chasing after it
4	Pench N.P.	[Video] The mother tiger got between her cubs (as well as food) and a sloth bear, and remained still. The bear stopped when it noticed the tiger about 8 m in front of it. The bear stood up. The two animals stood across from each other remaining relatively still. The bear stood up for a second time, and the tiger did not react. Finally, the bear ran off, away from the tiger and into the forest. The tiger did not follow
5	Bandahvgarh N.P.	[Video] A tiger returned to her den area and seemed aware that something was not right. She crept around the cave area, and finally a sloth bear charged out of a shallow rock den. They each swiped at each other with their front paws. The tiger retreated enough for the sloth bear to leave the cave and run down a hill a few feet. The bear turned back to the tiger which had pursued it. After some grunting, the bear turned and ran again, occasionally stopping and turning back to the tiger, which continued to slowly pursue the bear. This went on as the bear continued to run and then turn around and huff. The tiger eventually stopped pursuing, and the bear ran off
6	Tadoba N.P.	[Video] A sloth bear mother and cubs were retreating from a large male tiger. The mother bear realized the tiger was pursuing her and her cub, and she could not outrun the tiger. She turned around and charged the tiger into some bamboo. The tiger regroups, and the two fought in the bamboo with the tiger attempting to get a hold of the bear. The subadult cub whined and stood up and down in the background. The bear held its own in the early conflict. Whenever the bear was able, it righted itself and charged back into the tiger's face. The cycle continued with the bear pushing the tiger back a few feet, and then the tiger moving forward. The bear bobbed up and down, and the tiger moved back. The bear was careful to stay right in the tiger's face. The fight shifted to an open area, as some film was cut. The two wrestled, and there was not much movement. At one point it looked as though the tiger may have had the bear in a death grip around its neck, but the bear eventually broke free and chased the tiger. The bear pushed the tiger back into a waterhole, and eventually the tiger gave up and sats in the waterhole exhausted while the sloth bears scampered off
7	Nagarhole N.P.	[Photographs and Narrative] A small sloth bear charged out of the forest woofing at the large male tiger, which lied down unalarmed and looked at the bear. The bear approached the tiger within 1 m, checking out the tiger from all angles. The bear finally slipped back into the forest and disappeared. The tiger walked a few more meters and went to sleep
8	Tadoba N.P.	[Video] A sloth bear was walking through the woods and then running with three tigers chasing it. The tigers killed the bear
9	Ranthambore N.P.	[Video] A tiger was crouched and agitated. The tiger came to her feet and retreated several steps as a sloth bear charged into the picture; the bear stopped in front of the tiger. The tiger regrouped and held her ground as the bear took a few more steps forward. The tiger took a few steps forward and snarled. The bear stood up on two legs. The bear then dropped to four legs, and the tiger stepped forward, the bear stood back up
10	Tadoba N.P.	[Video] A tiger stalked a bear at night in the dark. Then the bear turned and charged. The bear stood up on two legs and then came back down as the two swatted at each other. The bear was the aggressor, moving the tiger backward. They disappeared into the darkness, and then the bear was walking back the direction from which it came. A few seconds later the tiger followed the bear again, and then the bear jumped back into the light at the tiger. They both held ground, and then the sloth bear moved off again. The tiger again started to follow, and again the bear jumped into the light at the tiger. Again, they both held ground for a few seconds. Then the bear moved away, and the tiger slowly followed. All was dark at the end of video, and we once again see the tiger holding ground, but we cannot see the bear. Notes to the video state that the bear and its cub were killed by the tiger, but there is no video of that
11	Ranthambore N.P.	[Video] A sloth bear was in a tree. The tiger attempted to get at the bear while the bear repositions itself and swats at the tiger. The tiger gave up and left the area
12	Kanha T.R.	[Video] A mother sloth bear with a cub on her back climbed up a steep bank by a road. A large male tiger was nearby. The tiger stalked the bear, and when the tiger got close the bear, the bear did not charge but whined loudly. There was a loud, short fight, and the tiger quickly killed the bear. The cub attempted to crawl away, but the tiger killed the cub
13	Ranthambore N.P.	[Video] A sloth bear stood up on two legs to face an approaching tiger. The tiger had already turned around once the bear stood up, and the bear charged and chased the tiger 50–100 m
14	Ranthambore N.P.	[Video] A tiger was lying on a two‐track road watching a large sloth bear. The bear charged the tiger, which stood up to move forward, but instead, the tiger turned and ran from the bear. The bear did not chase the tiger
15	Ranthambore N.P.	[Video] A tiger was stalking a bear. The bear noticed the tiger and took off running when the tiger was roughly 8 m away. The tiger did not chase the sloth bear
16	Tadoba N.P.	[Video] A tiger noticed a bear before the bear is on screen. The tiger crouched down to watch the bear walk right by within, what appears to be, roughly 5 m. The bear did not notice the tiger, and the tiger continued to watch as the bear walked by. After the bear drank at a waterhole, it started wandering off. When the bear got 15–20 m away from the tiger, the tiger started to follow. The tiger closed the gap between the two of them, and when the distance was down to about 12 m, the bear turned to see the tiger and it moved as though it was about to stand up, but it did not when it saw the tiger stop and sit on its haunches. The bear then calmly crawled up a bank and into a brushy area. The tiger did not move as the bear disappeared from sight. The tiger sat still for over a minute after the bear disappeared
17	Ranthambore N.P.	[Video] A sloth bear is approached a waterhole in which a tiger is lying. As the bear got closer to the waterhole, the tiger growled but did not move. The bear half stood up, but the tiger never moved. The bear eventually went around to access the waterhole from another direction and get a drink. The tiger stayed in the waterhole. The bear walked off
18	Ranthambore N.P.	[Video] A bear walked by a tiger, perhaps within 8 m. The tiger watched the bear without moving. Nothing came of the interaction. It appeared that the bear did not know the tiger was there
19	Ranthambore N.P.	[Video] A bear was walking through the woods (partially obscured). Suddenly the bear spun around and partially stood up while almost simultaneously huffing and charging. A tiger was right behind the bear (within 3 m). The tiger backed off and ran. The bear did not pursue the tiger
20	Tadoba N.P.	[Video] A sloth bear was walking to the river's edge and looked down from the high bluff to see a tiger lying in the water. The bear backed up and walked back into the forest. The tiger saw the bear about the same time that the bear saw the tiger, and after a second or two, the tiger jogged up the hill to chase the bear. The video stopped until the tiger returned to the river. The video states that the tiger came right back after chasing the bear off. The tigress then returned to the water
21	Ranthambore N.P.	[Video] A tiger was lying down and watching a sloth bear mother and two cubs walk by foraging. After about 1 min, the tiger got up and started to stalk the cubs, which are a small distance from the mother. The tiger suddenly stopped and looked off to her left, where the mother bear was (off camera). The tiger then started running off, and the camera followed the tiger, though shortly afterwards the mother bear came into the picture chasing the tiger. The tiger ran out of the area, but the mother bear did not follow the tiger far, as she stopped and turned toward her cubs that bounded to her
22	Tadoba N.P.	[Video] A sloth bear went to a river's edge to get a drink. A tiger was perched at the top of a bluff overlooking the river. The tiger began to get up and stalk the bear but quickly stopped. The tiger settled back down and watched the bear. The bear eventually left never knowing the tiger was there. As the bear left the area, the tiger got up to watch bear leave but never stalked it, but rather lied down again
23	Unknown	[Video] A large bear was walking along a dirt road in front of a jeep. Suddenly, beside a few trees, the sloth bear stood, huffed and began chasing a tiger, which is only seen in the video as it hastily retreated. The sloth bear chased the tiger over 20 m before the video ends
24	Unknown	[Video] A bear sprinted across a dirt road chased by a tiger that quickly gave up the chase
25	Nagarhole N.P.	[Video] A tiger either saw or heard a bear from the waterhole and got up to investigate. A sloth bear came out of a forested area and remained still while observing the tiger. The bear eventually started moving forward, toward the tiger. Just as the tiger crested the hill, the bear stood up on its hind legs and charged the tiger. The tiger held its ground for a few seconds before it ran off with the bear in pursuit. The bear pursued the tiger until it left the area
26	Nagarhole N.P.	[Photographs and Narrative] A sloth bear wandered upon a tiger at a waterhole. The bear chased off the tiger. The tiger returned, and once again the bear chased off the tiger. The tiger returned a third time, and the bear seemed to have moved on
27	Ranthambore N.P.	[Video] Two tigers (a mother and nearly grown cub) were moving slowly toward a sloth bear that was slowly backing off. The tigers roared. The sloth bear continued to back up and then stood several times. Finally, the sloth bear had backed up to a dirt road, and which point it turned and ran about 20 m before stopping to look back. The tigers did not follow
28	Tadoba N.P.	[Video] A tigress was walking down a two‐track. A bear came out from the surrounding forest and started walking down the same two‐track. The bear did not appear to notice the tiger walking in front of it. The tiger noticed the bear behind her and turned to face the bear in an unaggressive manner. The bear got very close to the tiger before noticing it was there, at which point the bear quickly stood up. The tiger seemed nonplussed and crouched down twitching her tail. The bear settles down to four legs and remained still. Neither the tigress nor the bear seemed overly stressed. The bear stood a second time, but the tigress did not react. After a not very intense stand‐off, the bear turned to notice the jeep behind him, actually turning its back on the tigress. After a few seconds, the bear ran off into the woods. The tigress watched in a very mellow state. She stayed lying down for a short period while licking herself, and then got up and continued to wander down the road as if nothing happened
29	Tadoba N.P.	[Video] A tiger was sitting on a concrete slab (perhaps a water structure), roughly 12 m from a sloth bear. The tiger sat up and stared at the sloth bear that was at the edge of a forest area. The sloth bear initially remained still and then took a few steps forward. The tiger seemed a little nervous and came to all fours. The bear walked back into the jungle and walked around the tiger's location from inside the woods. The tiger laid back down but kept watching the bear's movements. The bear circled all the way around the tiger and seemed to keep going. Nothing more came of the interaction
30	Sariska Tiger Reserve	[Video] A tiger was stalking a bear and surprised it from the front as the bear was digging at something (possibly termites). The bear stood up on its hind legs four times, while scooting a little backwards. The two animals swatted at each other with their front paws. The bear kept backing off slowly and appeared to run off at the end of the video. Further information of the encounter states that the tiger had two cubs and a goat carcass nearby and was likely just attempting to deter the bear from getting near its cubs and carcass
31	Southern India	[Video] In a distance a sloth bear chased a tiger. The tiger stopped and turned back to the bear that immediately went up on its hind legs. They swatted at each other, and the bear pushed the tiger backwards and started chasing it again. However, once again, the tiger turned back to the bear, which again stood up on its hind legs. This time the tiger did not turn and run but remained still. The bear stood a few more times, but the tiger did not move. The bear then started walking backwards until it was more than 3 m away from the tiger at which point it turned and ran. The tiger did not chase
32	Unknown	[Video] A sloth bear charged at a tiger at close quarters. The tiger jumped up and swatted at the bear. The tiger turned to run as the sloth bear started to stand and charge for a second time. The tiger turned to run away from the bear. It is an explosive encounter. The video lasted roughly 3 s
33	Unknown	[Video] A sloth bear family (mother and two cubs) was standing up and down toward a tiger they observed on the other side of some bushes. The tiger seemed a little unnerved but never got up nor ran away. The bear family unit eventually walked off
34	Ranthambore N.P.	[Video] A large tiger walked adjacent to a bear, not toward the bear, but was intently looking at the bear. The sloth bear did not move for several seconds. Finally, the bear charged at the tiger. The tiger did not give ground, and the bear stopped and stood. They then swatted at each other, but neither made significant contact with the other. They stopped swatting at each other, and the bear again stood as they each held their ground. The tiger appeared to back up a little, and then the bear began backing off
35	Ranthambore N.P.	[Video] A tiger was walking down a dirt road surrounded by forest. In the foreground, a sloth bear was climbing down from a tree, possibly because it saw the tiger coming down the road. The tiger initially did not seem to be aware of the bear. Once the bear climbed out of the tree it immediately charged the tiger. The tiger, unconcerned, watched the bear charge toward it, and finally, after the bear got very close, turned and ran. The bear continued to chase the tiger across a dry riverbed and up the embankment before the bear slowed down, though it kept following the tiger
36	Ranthambore N.P.	[Video] A tiger was crouched down on the road, and a sloth bear is on all fours roughly 3–10 m away (medium distance). They looked at one another but neither moved. The bear then stood up on two legs, but it did not elicit a response from the tiger. After about 5 s, the bear returned to all fours, and the standoff continued. After roughly 6 s, the tiger got out of its crouch and began walking directly at the bear. After a few seconds the bear turned and ran off the road and part way up a hill. After the bear ran about 4–5 m, it turned to face the tiger. The tiger began a slow trot when the bear began to run off but did not chase the bear off the road. At the end of the video the bear was still watching the tiger, which was still on the road no longer facing the bear
37	Unknown	[Video] A sloth bear was walking on a dirt road toward a crouching tiger it didn't notice. When the bear noticed the tiger, it stood up twice in quick succession. The tiger did not move from its crouching position and seemed to have no interest in engaging with the bear. After the bear stood twice, it looked to the side as if ready to move away from the tiger. At this point the video ended
38	Ranthambore N.P.	[Video] A sloth bear was in a scrubby forest area, and the view is partially obscured. The bear was looking at an off‐screen tiger. The bear stood and charged at a very large tiger. The tiger held its ground. The bear stopped in front of the tiger and stood up and down four to five times huffing at the tiger. The tiger growled loudly, but appeared to retreat a little as the bear continued to stand up and down huffing. The tiger growled a second time and appeared to walk away, outside of the video. The bear seemed to relax a little but remained still
39	Kanha N.P.	[Video] A tiger was following a sloth bear that is outside of the video. Suddenly the tiger stopped, and a sloth bear charged the tiger. The tiger turned and ran, and the sloth bear followed, ending the video
40	Jim Corbett N.P.	[Video] There are three different videos of this interaction, but there is not a full video. Notes in the video claim that the tiger eventually killed the bear. A large tiger approached a sloth bear from behind, which appears to already be injured. The bear turned and faced the tiger and then pushes the tiger backwards. In one other shot the tiger slowly crosses right in front of the bear, and the bear finally reacted by huffing loudly and pushing the tiger backwards
41	Nagarhole N.P.	[Video] A tiger was watching a sloth bear family unit (mother and two cubs). It sprinted at one of the sloth bears from a distance of roughly 7 m. It tackeled one of the cubs in long grass. The cub began fighting back while the mother and other cub ran over to join the fight. The tiger was overwhelmed by the bears and sprinted away with all three of the sloth bears giving chase. The cub that was attacked appeared to have an injured rear leg due to a limp
42	Tadoba N.P.	[Video] A young tiger approached a sloth bear. The sloth bear stood, barked loudly and took a few steps toward the young tiger, scaring it away, but did not chase the young tiger. The tiger stopped and then started moving back toward the sloth bear, at which point the sloth bear barked again and started chasing the young tiger. The tiger ran off into the woods. The sloth bear stopped chasing for a few seconds but then started barking again and running into the woods. A tiger cub walked into a clearing, and once again the sloth bear comes out of the woods chasing the tiger away. Notes on the video stated there were two cubs and a female tigress in the area. The event ended with the sloth bear chasing all of them off with no injuries to either party
43	Ranthambore N.P.	[Video] A bear walked toward a waterhole. It walked past a large tree, sniffing, and when it got to the other side of the tree, it saw a tiger lying in a waterhole 3–5 m away. The sloth bear froze and watched the tiger, which did not move other than twitching its tail. In a sudden burst, the sloth bear ran away from the tiger about 20–30 m before stopping on a dirt road to look back at the waterhole to see if the tiger was pursuing. After several seconds of the tiger not pursuing, the sloth bear continued to move away from the waterhole but walked in a more leisurely manner. The camera scans back to the tiger, which was still lying in the waterhole

**TABLE A2:** Sloth bear tiger interactions, initial reactions and result of interactions.

#	Does bear have cubs	# of tigers	Distance when bear observes tigers	How does bear initially react to tiger	How does interaction end
1	No	2	Close	Stand, huff, and charge (does this with each tiger independently)	Both tigers run off a short distance and hold ground. Sloth bear then runs off and is not followed
2	Yes (2 on back)	2	Close	Stand, huff, and charge (does this with each tiger independently)	Both tigers run off a short distance and hold ground. Sloth bear then moves off and is not followed
3	No	1	Close	Stand, huff, and charge	Tiger runs off
4	No	1 + 2 cubs	Medium	Stands and then remains still	Tiger makes no aggressive moves and the sloth bear runs off
5	No	1	Close	Swatting (backed up to den and rock wall so fights its way out)	Sloth bear runs off and tiger follows a short distance
6	Yes – 1 yearling	1	Medium	Runs – but turns and charges when tiger pursues	A long fight ensues that lasts over 10 min. Tiger eventually lets the bear run off, and exhausted, returns to the waterhole
7	No	1	Unknown distance	Charges and stands	Tiger watches the small bear (in amusement). Eventually the bear moves away
8	No	Three young adults	Medium	Runs	Tigers kill the bear
9	No	1	Medium	Charges – unknown if it stood first	Bear is standing and tiger is crouching
10	No	1	Unknown distance	During encounter stands and charges multiple times.	Reportedly the tiger killed the bear
11	No	1	Unknown distance	Bear in tree, does not climb higher but does turn in tree to face tiger	Tiger moves off and bear stays in the tree
12	Yes (1 on back)	1	Close	Remains still (loud vocalizations)	Tiger kills the mother bear quickly and then kills the cub
13	No	1	Close	Stand, huff and charge	Tiger runs off with the bear chasing
14	No	1	Medium	Charges – unknown if it stood first	The bear chases the tiger off
15	No	1	Medium	Runs	The bear runs off and the tiger does not pursue
16	No	1	Long	Looks and keeps walking	The bear walks off into the vegetation and the tiger does not follow
17	No	2	Medium	Remains still	Sloth bear walks around tiger to approach the waterhole from a different direction, gets a drink and leaves. Tiger watches
18	No	1	Bear never sees tiger	Bear never sees tiger	Sloth bear walks by and tiger just watches
19	No	1	Close	Stand, huff and charge	Tiger runs off, bear chases a short distance and stops
20	No	1	Medium	Remains still – Backs up into forest and moves away	Tiger pursues the sloth bear but quickly returns to the river
21	Yes – 2 yearlings	1	Medium	Mother charges – Unknown if the mother stood first	The mother bear chases the tiger off
22	No	1	Bear never sees tiger	Bear never sees tiger	Tiger watches the bear leave the area
23	No	1	Close	Charges (unknown if stood)	Tiger runs off with bear chasing
24	No	1	Unknown Distance	Bear runs by and after a second a tiger follows	Tiger stops chasing the bear
25	No	1	Long	Holds ground – then stands and charges	The bear chases the tiger off from the area
26	No	1	Close	Stand, huff, and charge	Tiger chased off but eventually returns to the area after the bear is gone
27	No	2 (mother and large cub)	Unknown Distance	A bear is being backed up by two tigers	The sloth bear runs off and the tigers do not follow
28	No	1	Close	Stands	The sloth bear eventually runs off and the tiger does not follow
29	No	1	Long	Holds ground – Walks around tiger in woods	Tiger watches as the bear moves around the tiger.
30	No	1	Close	Stands and Swats at tiger	Sloth bear runs off
31	No	1	Unknown distance	Sloth bear charging and then tiger holding ground and fighting	Several interactions before the bear is allowed to back off and run away
32	No	1	Unknown distance	Sloth bear charging	The tiger runs off
33	Yes (2 yearlings)	1	Close	All three bears Standing up and down, quickly and vocalizing	The tiger holds ground, and the bears eventually moves away
34	No	1	Medium	Remains still and then charges	The tiger and the bear are backing off from one another
35	No	1	Medium	Charges	The bear chases the tiger across a dry riverbed and up an embankment
36	No	1	Medium	Remains still, then stands, then remains still When tiger walks at the bear, the bear runs up hill 4–5 m and turns back to tiger	The tiger seems unwilling to chase the bear, but the bear is still watching the tiger
37	No	1	Close	Stands twice	Crouching tiger does not react and the bear moves off
38	No	1	Close	Stands and charges	The bear and tiger both start to back off
39	No	1	Medium	Charges – It cannot be seen if it stands first	Sloth bear chases tiger off
40	No	1	Unknown distance	Bear lying in leaves gets up to charge tiger (unclear if the bear has already been injured)	The video states that the bear was killed
41	Yes – 2 walking	1	Medium	Mother bear and one cub charge – The cub that is charged by tiger starts to run before being pounced on	The mother and other cub attack the tiger, and the tiger runs off with all three sloth bears in pursuit
42	No	3 (Tigress and two older cubs)	Unknown distance	The sloth bear barks and stands. From the description of the event, it is uncertain if this is the first interaction	The tigers run away after being chased multiple times
43	No	1	Medium	Remains still and watches the tiger, which is sitting in a waterhole	In a sudden movement, the bear turns and runs 20–30 m. It then stops and looks back to see if it is being followed. The tiger does not leave the waterhole. The bear then walks away back into the forest
